# Corrigendum: Prevalence of post-traumatic stress disorder status among healthcare workers and its impact on their mental health during the crisis of COVID-19: A cross-sectional study

**DOI:** 10.3389/fpubh.2022.1001246

**Published:** 2022-08-10

**Authors:** Yue Yang, Di Liu, Bingshuo Liu, Weiyan Ou, Licheng Wang, Yuanshuo Ma, Lihua Fan, Yu Shi, Lei Shi

**Affiliations:** ^1^Harbin Medical University Cancer Hospital, Harbin, China; ^2^School of Marxism, Harbin Medical University, Harbin, China; ^3^School of Health Management, Southern Medical University, Guangzhou, China; ^4^School of Health Management, Harbin Medical University, Harbin, China; ^5^Vanke School of Public Health, Tsinghua University, Beijing, China

**Keywords:** COVID-19, PTSD, healthcare providers, self-efficacy, mental health

In the published article, there was an error in affiliation 1. Instead of “Tumor Hospital Affiliated of Harbin Medical University, Harbin, China,” it should be “Harbin Medical University Cancer Hospital, Harbin, China.”

The authors apologize for this error and state that this does not change the scientific conclusions of the article in any way. The original article has been updated.

## Publisher's note

All claims expressed in this article are solely those of the authors and do not necessarily represent those of their affiliated organizations, or those of the publisher, the editors and the reviewers. Any product that may be evaluated in this article, or claim that may be made by its manufacturer, is not guaranteed or endorsed by the publisher.

